# Evaluation of Nonproximal Caries as Predictor of Proximal Caries in Primary Molars

**DOI:** 10.5005/jp-journals-10005-1557

**Published:** 2018

**Authors:** Vineet Dhar, Soe Mon, Mark D Macek

**Affiliations:** 1 Department of Pediatric Dentistry, University of Maryland School of Dentistry, Maryland, USA; 2 Department of Pediatric Dentistry, Severn Pediatric Dentistry, Maryland, USA; 3 Department of Dental Public Health, University of Maryland School of Dentistry, Maryland, USA

**Keywords:** Caries, Nonproximal lesions, Proximal lesions

## Abstract

**Background:**

Most caries prevalence studies are conducted in community-based settings, usually with no radiographs, therefore, it is questionable if visual examination alone captures the true extent of disease.

**Aim:**

Since it is relatively easy to diagnose occlusal and facial/lingual surface (nonproximal caries) on visual examination, our aim was to evaluate for an association between nonproximal caries (NP) and proximal caries (P), which if present can provide a tool to help decision makers in estimating the true extent of the disease.

**Design:**

A cross-sectional retrospective chart audit was done using records of 106 children to determine the association between NP and P caries in the primary dentition.

**Results:**

Our mean dft for NP lesions only was 1.55. Based on our data, the mean dft considering all pit and fissure plus the proximal lesions was 2.54, which is a 63.2% increase from the dft based on NP caries only. We found a significant association between NP caries and radiographically detectable P caries. Proximal lesions were twice as likely to exist on primary molars when non-proximal/pit and fissure carious lesions were present.

**Conclusion:**

The results imply that proximal caries, and thus need for treatment, are being underestimated during visual exams.

**How to cite this article:**

Dhar V, Mon S, Macek MD. Evaluation of Nonproximal Caries as Predictor of Proximal Caries in Primary Molars. Int J Clin Pediatr Dent, 2018;11(6):457-461.

## INTRODUCTION

As per the 2011 to 2012 National Health and Nutrition Examination Survey (NHANES), 22.7% of children ranging from 2 to 5 years of age had one or more primary teeth affected by dental caries, and 55.7% of children had one or more primary teeth affected by age 6–8. In the permanent dentition, 13.8% of children aged 6–8 had dental caries, 28.8% of children were affected by age 9–11 and 50.1% of children aged 12–15 had dental caries.^1^

Diagnosis of dental caries is usually based on a thorough clinical examination and/or radiographic findings. Since proximal caries can be difficult to diagnose clinically, bitewing radiographs are routinely recommended for children with varying caries risk to aid in the detection of early proximal lesions during clinical examinations.^2^ If radiographs were not used in the clinical setting, undiagnosed proximal caries would remain untreated, and in addition to causing pain and discomfort to the child, it may increase the risk of developing proximal caries on the permanent teeth.^3,4^

Proper diagnosis of dental caries is also key for accurately recording dental caries prevalence at the population level. It is doubtful that the visual screening examination that accompanies epidemiological surveillance alone captures the true extent of disease. Data related to the prevalence of dental caries is routinely collected and reported both state and national level. This helps the policy makers understand the burden of disease, analyze existing disparities and treatment needs at community levels. Since most caries prevalence studies are conducted in community-based settings, usually with no radiographs, it is likely that some proximal caries lesions are underreported.

American Academy of Pediatric Dentistry (AAPD),^5^ suggests that the presence of existing caries is said to be the best predictor of caries. Since it is relatively easy to diagnose existing pit and fissure caries on occlusal and facial/lingual surfaces (nonproximal caries) on visual examination, it will be useful to know if there exists any association between nonproximal (NP) and proximal caries (P).

Such an association, if present, can provide a tool to help epidemiologists estimate the true extent of the disease. We hypothesized that the number of decayed teeth detected by visual examination alone underestimates the true extent of the disease.

To test this hypothesis, we conducted a cross-sectional pilot study with the aim to identify the extent to which dental caries surveillance in population-based studies underestimates the true extent of disease. These analyses will give policymakers a more accurate view of the needs of the community. An accurate estimation of health care needs is critical for proposing, estimating costs implementing preventive policies, and meeting the restorative care needs at all levels of society.

## MATERIALS AND METHODS

This cross-sectional study involving a chart audit was carried out in the division of pediatric dentistry, University of Maryland School of Dentistry. The research was approved by the Institutional Review Board, University of Maryland, Baltimore. Electronic patient records such as dental charts and radiography software were reviewed to obtain data in this study. Using the electronic records, we were able to review clinical and radiographic findings recorded in the chart at the time of their first visit to our dental clinic.

**Figs 1A and B:**
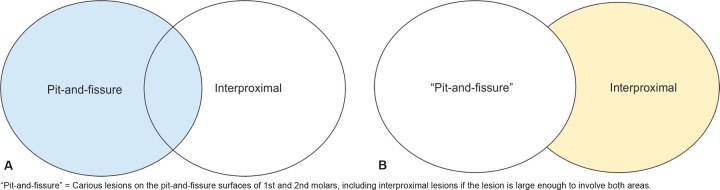
NP/Pit- and- fissure caries category criteria

**Fig. 2:**
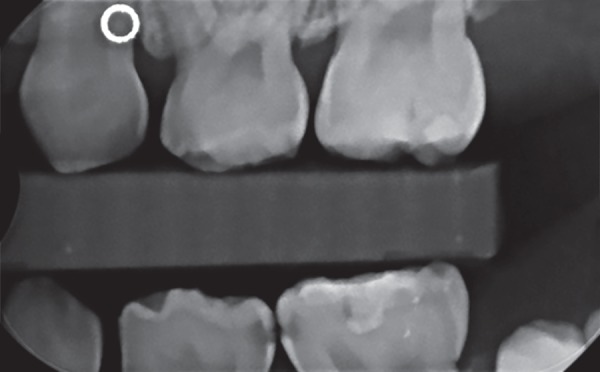
Example of P caries on teeth # K and L

For the purposes of this study, pit- and- fissure carious lesions on the occlusal/facial/lingual surfaces of the primary molars, as recorded on the charts after clinical and/or radiographic examination, were categorized as NP caries. We also included in this category, any carious lesions that based on chart entry or radiographic assessment could have been visually detected. Therefore, the NP/pit- and- fissure category included large proximal lesions extending to other surfaces that would be clinically visible (Fig. 1). Proximal caries in primary molars was defined as mesial and/or distal caries lesion extending to the dentin-enamel junction and beyond as recorded solely from bitewing radiograph of posterior teeth. Such a lesion would not have been detected solely on the basis of visual examination (Fig. 2). Due to variation in diagnosing incipient enamel lesions during the calibration exercise, incipient enamel proximal lesions were not considered in this study.

### Inclusion Criteria

Electronic patient records with:

- One or more Bitewing radiograph (BWX) ADA procedure codes entered (D0270, D0272, D0273, D0274, D0277)
- Treatment date between 1/1/2010–3/3/2014 of patient age ≥ 36 months and ≤84 months at the time of treatment

### Exclusion Criteria

- No radiographs or non-diagnostic radiographs such as interproximal overlapping, a region of interest is not covered and poor contrast
- Children with special needs/complex medical problems
- Children with bitewing radiographs and/or treatment done elsewhere before visiting the dental school

Sample size calculation: There were a total of 1,718 patients fitting these criteria having 938 males 780 females. To detect a 20% point difference between proximal and nonproximal caries groups, a sample of 206 radiographs were needed to be reviewed. Given these numbers, and intention for this project, a sample of 212 radiographs was considered reasonable for this project.

The charts and radiographs were screened by a single examiner (SM) using convenience sampling. Approximately 20 radiographs were evaluated by two additional evaluators (VD and MDM) to verify that the examiner's scoring criteria for NP and P lesions were as per the definition. The number of mesial and distal caries on a radiograph in primary dentition were recorded. The number of nonproximal carious lesions were recorded by charted notes/findings and radiographs.

The following outcomes were studied:

- *Result group 1:* Presence of both P caries and NP caries.
- *Result group 2:* Presence of P caries but no NP caries.
- *Result group 3:* Presence of NP caries but no P caries.

The data was entered onto Microsoft Excel spreadsheets and examined for any data entry errors. A password protected computer was used to store this data. Statistical analysis was using SAS for Windows 9.3 (SAS Institute, Inc., 2002–2010). The mean number of P and NP caries was calculated. The association between P and NP caries was evaluated using chi-square analysis and odds ratio.

### RESULTS

A total of 212 radiographs taken from records of 106 children were included in the study, out of which 54 (50.9%) were boys and 52 (49.1%) were girls and all of these children were on Medicaid. The mean dft of all children with NP carious teeth was 1.55 and with P caries was 1.61 (Table 1).

**Fig. 3:**
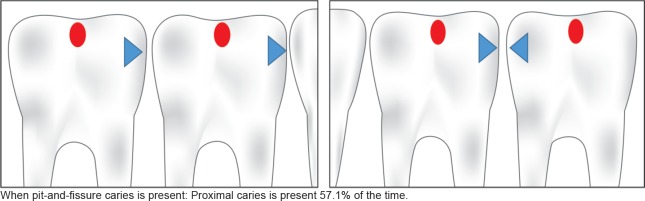
Association of presence of NP caries with P caries (all molars data)

**Fig. 4:**
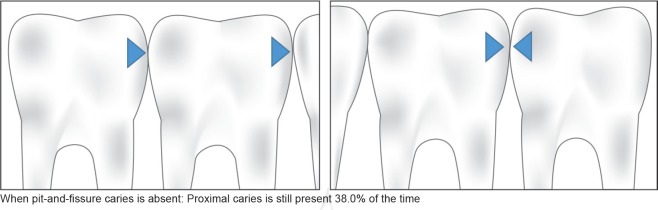
Association of absence of NP caries with P caries (all molars data)

On reviewing the data from both first and second primary molars, we found that the child with pit- and- fissure carious lesions on occlusal/buccal/lingual surfaces had 2.18 (1.00–4.74) times greater chances of having proximal carious lesions when compared to a child with no pit- and- fissure caries. We observed that when pit- and- fissure caries was present, proximal caries was present 57.1% of the time (Table 2 and Fig. 3). Interestingly, when pit- and- fissure caries was absent, proximal caries was still present 38.0% of the time (Table 2 and Fig. 4). A direct implication of this finding is that in a field-based study, where clinical exam shows no caries, we still have 38% chances of having proximal caries, which is probably not reported in a routine visual exam.

Our mean dft for NP/pit-and-fissure lesions only was 1.55. Based on our data, the mean dft considering all lesions (pit and fissure plus the proximal lesions) was 2.54, which is a 63.2% increase from the dft based on NP caries only. Hence, we cannot rule out the possibility that the current caries prevalence is underreported.

Based on our results, we found a significant association between NP caries and radiographically detectable P caries. Specifically, proximal lesions, which would be difficult to detect on visual examination, were twice as likely to exist on primary molars when non-proximal/pit- and- fissure carious lesions were found (OR 2.18). We, therefore, accepted the hypothesis that the number of decayed teeth detected by visual examination alone underestimates the true extent of the disease.

### DISCUSSION

Authors carried out this cross-sectional study to evaluate the presence of NP and P caries and look for any possible association between both in the primary molars. This is a pilot study that allows us a glimpse of the possible underreporting of the prevalence of dental caries. We understand that there is a need for further research and perhaps randomized clinical trials are needed to substantiate our findings.

In our methodology, we defined nonproximal caries lesions as carious lesions on the pit-and-fissure surfaces of 1st and 2nd molars, including interproximal lesions if the lesion is large enough to involve both areas. However, as per our data, we estimate that only 4.0% of the time would a proximal lesion be visualized when a nonproximal is detected because the occlusal decay is so extensive (e.g., a bombed-out tooth).

**Table 1:** Demographic Data

*N (radiographs)*		*212*	
Number of children		106	
Boys	54 (50.9%)	Girls	52 (49.1%)
Mean dft of all children with nonproximal carious teeth	1.55	Mean dft of all children with proximal carious teeth	1.61
Mean dft of all children with nonproximal carious and proximal carious teeth		2.54	

**Table 2:** Presence or absence of NP and P carious lesions on all primary molars

*Nonproximal lesions*	*Proximal lesions*		*Total*
	*Present*	*Absent*	
Present	32 (57.1%)	24 (42.9%)	56 (52.8%)
Absent	19 (38.0%)	31 (62.0%)	50 (47.2%)
Total	51 (48.1%)	55 (51.9%)	106 (100.0%)

Odds ratio = 2.18; NP: Nonproximal; P: Proximal

As per the AAPD caries risk assessment tool, caries risk can be determined on basis of several biological, protective and clinical factors.^5^ Previous caries experience, parental education, socioeconomic status and mutans streptococci levels are among the reliable predictors for new caries.^6,7^ Since caries experience is one of the best predictors for new caries,^5^ it was of interest to know if existing NP caries can serve as reliable objective criteria to predict the presence of P caries that may or may not be visible clinically.

A 7th-year longitudinal clinical study done on 6–8 years old children reported that caries experience in the primary teeth and maternal educational level were good predictors for new lesions in the permanent dentition. ^8^In our study, we found that the likelihood of having proximal caries in primary dentition was 2.18 times higher if nonproximal lesions were present. One study reported that in their findings solely on the basis of clinical judgment, the number of carious proximal surfaces was 62.7% in males and 58.9% in females. On including bitewing radiographic examination, the numbers increased to 80.6% in males and 71.9% in females.^9^Another similar study reported that approximal caries identification in primary molars increased statistically with the bitewing examination. They found that out of the 25% lesions detected with the radiographic exam, 84.6% were enamel lesions, and 10.8% were dentine lesions.^9^ Yet another study reported that visual-tactile technique could detect only 43% of proximal caries compared to 91% for bitewing radiography.^10^ All these studies reinforce the need for bitewing radiographs to better detect proximal caries. Our study reports on the likelihood of detecting proximal caries when nonproximal caries is present in form of odds ratio (OR 2.18). We calculated a mean dft of 1.55 based on NP caries only and a mean dft of 2.54 based on all NP and P caries, signifying a 63.2% increase from the dft based on NP caries only.

Our study might be conservative in estimating the association between proximal and nonproximal caries since we did not include incipient enamel proximal caries in the analysis. We found it difficult to calibrate reliable identification of such lesions on radiographs due to the differences in interpretation of initial lesions and confounders like artifacts on radiographs and morphological variations. Therefore, it was decided to err towards being more conservative as against over diagnosing proximal lesions that may not actually exist. Despite the conservative approach, our results strongly supports the association between nonproximal and proximal caries.

Our results suggest that if nonproximal caries is not detected, there is a high 62% chance that there is no proximal caries and a 38% likelihood that proximal caries may still be present. This data, when substantiated with further research, is likely to have a considerable impact on the current understanding on the burden of the disease, following which, the policymakers may need to redirect efforts with an emphasis on preventive strategies and the need for establishing dental homes on a timely basis.

The current study had several limitations. We included a relatively large age range (3–7 years) to obtain the necessary sample size but the child's birthdate was not captured, and age-wise stratification was not done. This is an important variable since it is directly related to the time the tooth is at risk for developing caries. Though all included children were on medicaid and lived in a water-fluoridated community, we did not capture other caries risk factors such as socioeconomic status, water fluoridation, oral hygiene in our analysis. Since we conducted a tooth-level analysis, age-wise stratification, and inclusion of other child-level risk factors were beyond the intent of our analysis. These factors will be critical and should be included in a larger and more comprehensive prospective clinical study to evaluate this association. Being cross-sectional, the results of our study may not be generalizable or representative of the entire population. The findings of this study imply that the presence or absence of nonproximal caries can be used as a predictor for proximal caries, which may or may not be visible during a clinical exam. We consider our findings as preliminary that may be used to support further research in this area. However, considering the possible impact of our results, there is a definite need for a well-designed clinical study to confirm the true extent of association.

## CONCLUSION

- In the presence of nonproximal caries, the chances of the child having proximal caries are doubled.
- In absence of nonproximal carious lesions, a little over one-third proximal surfaces were still carious.
- Proximal caries, and thus need for treatment, are likely being underestimated during visual examinations alone.

### Why this Paper is important to Pediatric Dentists?

- The paper substantiates that epidemiological surveillance alone inadequately captures the true extent of dental caries in primary teeth of children.
- The paper shows that the presence of visible/non-proximal caries can suggest an increased possibility of the child having proximal lesions that may typically not be visible on clinical examination.
- Clinicians can use the possible association between proximal and visible/nonproximal caries as one of the factors to consider, while making a decision to take or not to take a diagnostic radiograph at a recall visit.
